# Predictive value of TRPV2 expression from peripheral blood mononuclear cells on the early recurrence of atrial fibrillation after radiofrequency catheter ablation

**DOI:** 10.1186/s12872-022-02992-0

**Published:** 2022-12-13

**Authors:** Xuebin Ling, Jun Wang, Xue Qin, Chufen Lin, Wei Jie, Yane Chen, Dajia Fu, Yang Yang, Qingwen Meng, Jing Lin, Hui Liu, Tianfa Li, Junli Guo

**Affiliations:** 1 Key Laboratory of Tropical Cardiovascular Diseases Research of Hainan Province, Cardiovascular Diseases Institute of the First Affiliated Hospital, Department of Cardiovascular Surgery, the Second Affiliated Hospital of Hainan Medical University, Key Laboratory of Emergency and Trauma of Ministry of Education, Hainan Medical University, Haikou, 571199 China; 2 Department of Health Medicine, Affiliated Haikou Hospital of Xiangya Medical College, Central South University, Haikou, 570208 Hainan China; 3 Department of Anatomy, School of Basic Medicine and Life Science, Hainan Medical University, Haikou, 571199 Hainan China

**Keywords:** TRPV2, Peripheral blood mononuclear cells (PBMCs), Early recurrence, Atrial fibrillation

## Abstract

**Background:**

Recent study has shown that the transient receptor potential vanilloid 2 (TRPV2) channel was exclusively upregulated in patients with atrial fibrillation (AF), and that this overexpression might be detrimental for occurrence and maintenance of AF. We aimed to characterize the expression levels of TRPV2 mRNA in peripheral blood mononuclear cells (PBMCs) with/without early recurrence of atrial fibrillation (ERAF) after radiofrequency catheter ablation (RFCA), and to find a reliable predictor for ERAF.

**Methods:**

65 patients of AF, who underwent RFCA successfully, then divided into two groups according to ERAF during following 3 months. PBMCs were isolated from whole blood by Ficoll gradient centrifugation before and after RFCA. Gene set enrichment analysis was performed to evaluate TRPV channels expression levels and Kyoto Encyclopedia of Genes and Genomes (KEGG) mapping was used for pathway enrichment analysis.

**Results:**

There was no significant difference in the TRPV2 mRNA expression level between the two groups before RFCA, while without ERAF group of TRPV2 expression was markedly reduced compared to ERAF group after RFCA. Moreover, the number of TRPV2 expression was confirmed as an independent predictor for the first time through receiver operating characteristic and Kaplan–Meier survival curve analysis. It should be pointed out that the above results were only used to predict ERAF, and have no predictive significance for late recurrence of atrial fibrillation according to the current data. Additionally, ERAF was inversely correlated with P wave dispersion. KEGG mapping further clustered 41 pathways, revealing that ‘‘cyclic guanosine monophosphate-protein kinase G signaling pathway’’ was significantly enriched.

**Conclusions:**

We firstly assume that downregulated expression of peripheral TRPV2 appear in patients without ERAF after RFCA. TRPV2 may thus represent a novel predictor of early phase after successful radiofrequency ablation.

**Supplementary Information:**

The online version contains supplementary material available at 10.1186/s12872-022-02992-0.

## Background

Atrial fibrillation (AF) can lead to heart failure, stroke, cognitive decline, dementia, and renal function injury, all of which seriously threaten human health. Currently, a conservative estimate of the adult prevalence rate of AF is 2–4% [1]. Radiofrequency catheter ablation (RFCA) should be widely used in the treatment of AF; however, early recurrence (ER) after initial successful pulmonary vein isolation (PVI) is common and is observed in 50% or more of patients [2, 3]. ER is considered to have a transient stimulatory effect (i.e., inflammation, temporary autonomic imbalances, and time-course of lesion formation), and ER represents the vital predictive value of developing late recurrence (LR) [4]. Many predictors of ER have been identified, such as older age, male sex, presence of structural heart disease, longer AF duration, nonparoxysmal AF type, higher CHA_2_DS_2_-VASc scores, larger LA size, C-reactive protein, and homocysteine level [3]. Meanwhile, ER following RFCA for paroxysmal AF (ParAF)/persistent AF(PerAF) is strongly associated with LR beyond the blanking period [5, 6]. Moreover, genetic variants of the AF-related gene have also been suggested to be associated with AF recurrence [7, 8]. Although there are many predictors of early recurrence of atrial fibrillation (ERAF), their lack of specificity and limited predictive value inhibit their clinical application. Therefore, novel specific predictors need to be developed urgently.

Ca^2+^ homeostasis plays an indispensable role in the maintenance of normal cardiac conduction and systolic cardiac function. Moreover, dysregulation of Ca2^+^-transporting transient receptor potential (TRP) channels is known to be the cause of the occurrence and recurrence of AF [9]. The transient receptor potential vanilloid (TRPV) family consists of six mammalian members, TRPV1–TRPV6, whose function is to permit the influx of Ca^2+^, along with Mg^2+^, Zn^2+^, and Na^+^ [10]. The TRPV family has emerged as a novel and interrelated system to detect and respond to various environmental stimuli, including mechanical, thermal, or chemical stimuli. Given this function, the TRPV family is considered to serve as sensor for monitoring specific responses to different exogenous and endogenous chemicals and physical stimuli [11]. It has been widely reported that TRPV2 mRNA is expressed in human peripheral blood mononuclear cells (PBMCs) and may represent a potential novel biomarker in various diseases (i.e., coronary heart disease [12], asthma [13], and inflammatory bowel disease [11]), as well as a prospective prognostic marker and potential novel therapeutic target for cancer treatment [14]. Furthermore, Irfan et al. [15] observed that TRPV2 gene expression is upregulated in the leukocytes of patients with nonvalvular AF and implied that TRP channels may be effective targets for the prevention or prophylaxis of nonvalvular AF. Recent studies have shown that Ca^2+^ overload could cause recurrence of AF, regulated by the TRPV2 channel, which could lead to an increase in intracellular Ca^2+^. TRPV2 mRNA from PBMCs is also highly expressed in nonvalvular AF, although it is unknown whether its expression changes after RFCA. Taken together, these findings suggest that PBMCs are in contact with multiple stimuli within the blood that have the potential to release TRP; thus, TRP may represent a crucial functional protein involved in the occurrence and development of AF and other diseases. However, no previous study has reported the relationship between TRPV2 expression in PBMCs and patients with AF after RFCA.

Here, we focused on the expression profiles of TRPV2 mRNA in PBMCs obtained from patients with AF after RFCA. We also examined the relationship between TRPV2 mRNA expression and ER, as well as other laboratory parameters. This study is the first to explore the expression of TRPV2 in patients with AF after RFCA and its significance in early recurrence of AF, which may provide clues regarding the disease pathway.

## Materials and methods

### Study population

Sixty-five patients with nonvalvular AF who were treated at the First Affiliated Hospital of Hainan Medical University were enrolled from May 2019 to May 2022. The patients’ baseline characteristics, procedural findings and follow-up data were collected prospectively and recorded in a computerized database. All of the patients demonstrated restored sinus rhythm after RFCA. ERAF was defined as a recurrence of AF after > 30 s during the first 3 months through in-hospital electrocardiogram(ECG) recordings, external resting and Holter ECGs. All of the enrolled patients with ERAF had AF without atrial flutter or atrial tachycardia. LR was defined as any recurrence of AF > 30 s after at least 3 months [16]. The exclusion criteria were valvular heart disease with moderate to severe stenosis or insufficiency of the cardiac valves, existing LA appendage thrombi, heart failure, coronary artery disease, dilated cardiomyopathy, hypertrophic cardiomyopathy, mental disorder, thyroid disorder, hepatorenal insufficiency, pregnancy, cancer, and patients who failed to cooperate with follow-up.

### Measurement of P wave dispersion (PWD)

All the participants underwent a routine standard 12-lead body surface ECG at admission and after RFCA, recorded at a paper speed of 25 mm/s. The junction between the isoelectric line and the start of P wave deflection defined as the onset of P wave, and the junction between the isoelectric line and the end of the P wave deflection defined as the offset of P wave. The PWD, as a marker of prolonged atrial conduction time, was calculated as the difference between the maximum and minimum P wave duration from the 12-lead ECG after sinus rhythm conversion, and mean PWD (mPWD) was defined as the average of the maximum and minimum P wave duration. The follow-up after radiofrequency ablation included a daily recheck of ECG in the hospital and routine ECG examination at 1, 3, 6, and 12 months after discharge. In the case that any of the patients experienced symptoms of chest tightness and palpitation, ECG examination was performed at any time. These data were independently measured by two ECG diagnostic physicians through the Nalong system (Nalong technology, Inc., China).

### Cardiac computed tomography (cCT)

All patients underwent cardiac cCT [a Dual Source (2 × 64 slice) CT (Somatom Definition Flash, Siemens, Germany)] before the procedure to assess pulmonary vein anatomy. cCT imaging raw data was imported in the segmentation software syngo.via (Siemens, Germany). Two radiologists measured the anterior and posterior diameters, left and right diameters, upper and lower diameters of the left atrium in diastole, and the anterior and posterior diameters of the pulmonary veins in cross section. The left atrial volume index (LAVI) is the product of (left atrial anteroposterior diameter × left–right diameters × upper-lower diameters)/body surface area(BSA).

### Echocardiogram

All patients underwent trans-thoracic echocardiograph (Philips EPIQ 7C, Netherlands) prior to RFCA, and a few patients have undergone cardiac ultrasound reexamination after RFCA. Recommendations of the American Society of Echocardiography were followed to measure echocardiographic indexes. The left atrial diameter (anterior–posterior, left–right diameters) was obtained in the parasternal long axis view during end-systole, and left atrial area was calculated by the product of “anterior–posterior diameters” × “left–right diameters”. Left ventricular ejection fraction (LVEF) was measured by modified Simpson’s method. Late diastolic peak velocity of mitral inflow(A) and early diastolic peak velocity of mitral inflow(E) were measured, which used to calculate the E/A ratio. We measured them from the mean value of 10 cardiac cycles during AF state. Trans-esophageal echocardiography was performed to exclude intracardiac thrombi.

### RFCA procedures

Prior to the procedure, all patients received oral anticoagulation (warfarin or rivaroxaban) for at least 1 months, and rule out the presence of LA thrombus by transesophageal echocardiogram within 24 h before RFCA. During the procedures, heparin was administered as continuous pumping at an intraprocedural activated clotting time > 300 s. PentaRay (Biosense Webster, Inc., Diamond Bar, CA, USA), a star shaped mapping electrode, was sent to the left atrium for modeling and matrix mapping after successful puncture of atrial septum under the guidance of a Carto 3 electroanatomic mapping system (Biosense Webster, Inc.). After modeling, the cold saline perfusion ablation catheter STSF (Biosense Webster, Inc., Diamond Bar, CA, USA) was sent to the left atrium for the positioning of the pulmonary vein, and the power mode was selected for ParAF ablation under the guidance of ablation index (AI). The parameters were: posterior wall power 45 W, AI = 380; top and bottom power 45 W, AI = 400; anterior wall power 50 W, AI = 450. The left and right pulmonary veins were isolated along the vestibular potential. For PerAF, in addition to the ablation line of ParAF, the left atrial roof line ablation (power: 45 W, AI = 400) and the mitral isthmus line ablation (power: 50 W, AI = 500) were performed. If cavotricuspid isthmus (CTI)-dependent atrial flutter was induced during the procedure, the cavotricuspid isthmus line (power: 40 W, AI = 400) and the atrial flutter originating from the superior vena cava were performed for superior vena cava isolation (power: 40 W, AI = 400). The stimulation mapping of corresponding positions was performed under sinus rhythm pacing to verify whether the left atrial roof line, mitral isthmus line and cavotricuspid isthmus line were successfully blocked. Successful RFCA was defined as exit block in pulmonary veins and entrance block in left atria was reconfirmed 30 min after PVI, as well as no atrial arrhythmia was induced after procedure. The maps of RFCA were included in Additional file 1: Fig. S1. If PerAF patients didn′t restored sinus rhythm after RFCA, a synchronized direct-current cardioversion should be applied and the energy should be set to 50–100 J.

### Blood samples

Peripheral blood samples were collected from patients before radiofrequency ablation of AF and then again when all patients recovered sinus rhythm at the end of radiofrequency ablation. All blood samples were collected by centrifugation of chemistry tubes. PBMCs were acquired through Ficoll gradient centrifugation of complete blood tubes followed by two washes with phosphate-buffered saline (PBS).

### Real-time PCR

RNA from PBMCs was extracted using the high pure RNA isolation kit (OMEGA, USA) and transcribed by Verso RT-PCR kits (TIANGEN, China). Quantitative PCR was performed using the SYBR Green SuperReal PreMix Plus qRT-PCR Kit (TIANGEN, China). The primers used were as follows: human TRPV2: forward, 5′AGTCAGTGCCCATGGA3′ and reverse, 5′GCCGATGGTGAATTTGAAGAG3′; human β-actin reference gene: forward, 5′CTGGAACGGTGAAGGTGACA3′ and reverse, 5′CGGCCACATTGTGAACTTTG3′. Each sample was measured three times, and the relative quantitative of TRPV2 mRNA expression was calculated using the 2^−△△Ct^ method (versus β-actin). All data were analyzed by the Applied Biosystems SDS 2.3 software using the threshold cycle (Ct) relative quantification method with β-actin as an endogenous control.

### Functional analysis of differentially expressed genes (DEGs)

To obtain a global view of the TRPV expression, hierarchical cluster was constructed to characterize changes across 10 samples [17] from GEO database. These samples were from the left atrial appendage tissues, including 5 sinus rhythm patients and 5 PerAF patients undergoing mitral valve surgery. There was no difference in atrial diameter, left ventricular function, medication and age between the two groups. Transcriptional profiling of heart tissue of AF patients was performed with GeneChip-Human Genome-HGU 133-Plus 2.0 arrays (Affymetrix, Santa Clara, California) [17]. Pathway enrichment analysis is used to identify functional categories of differentially expressed genes through the Database for Annotation, Visualization and Integrated Discovery (DAVID) (https://david.ncifcrf.gov/). The function charts of DEGs are built by using DAVID. KEGG (Kyoto Encyclopedia of Genes and Genomes) pathway enrichment analyses were applied for the DEGs, with the threshold of *p* value < 0.05. The rich factor is calculated as the ratio of the numbers of DEGs enriched in this pathway, to the numbers of all genes annotated in the same pathway. The Q-value is the corrected P-value with threshold < 0.05. Heatmap and scatter were plotted by https://www.bioinformatics.com.cn, a free online platform for data analysis and visualization.

### Statistical analysis

The numerical variables with normal distribution are expressed as the mean ± standard deviation. The t-test was used for intergroup comparison. The numerical variables that did not obey the normal distribution are represented by the median (25th and 75th percentile). The nonparametric rank sum test was used for intergroup comparison. Categorical variables are expressed as the number of cases (%) and the χ2 test for comparison between groups. Correlation was tested by two-tailed Spearman analysis. Binary logistic regression analysis was used to screen out the influencing factors of the indexes with statistical significance. Factors with a *p*-value < 0.1 on univariate analysis were considered in the multivariate model. Time to recurrence and event-free survival curves were analyzed using the Kaplan–Meier method with a 3-month ERAF and log-rank significance testing. The predictive effectiveness evaluation of influencing factors is expressed by the ROC curve. Missing values were minimal (except in the case of the ECG parameters after RFCA) and roughly equivalent between groups for all variables and were thus omitted. Statistical analysis was performed using SPSS version SPSS 26.0 (IBM Inc., Armonk, NY, USA), and significance was set at *p*-value < 0.05.

## Result

### Differentially expressed genes (DEGs) in response to TRPV channels

**Fig. 1:**
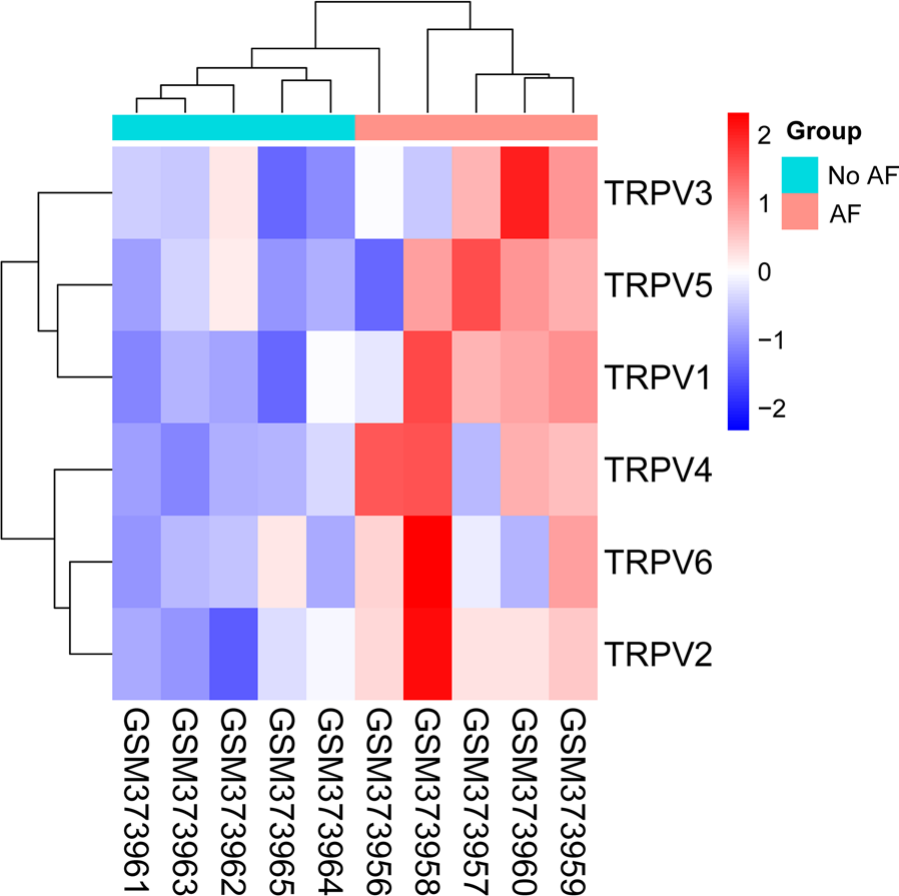
The heatmap of the differentially expressed TRPV channels genes by using heatmap package. The samples (GSM373961-65) were obtained from LA tissue of without AF individuals, and the other samples (GSM373966-70) were obtained from LA tissue of AF individuals. The data comes from GSE14975 of GEO database. Hierarchical clustering of DEGs was generated, and the expression levels were visualized and the scale from least abundant to highest range is from − 2.0 to 2.0. Their phylogenetic relationships were shown on the left tree. The top tree indicated the cluster relationship of the samples

By comparing individuals with and without AF through Wilcoxon tests, it was found that the TRPV1, TRPV2, and TRPV4 mRNA expression from the LA tissue of individuals with AF was significantly upregulated (*p* = 0.016, 0.0079, and 0.016, respectively). However, the TRPV3, TRPV5, and TRPV6 expression was not statistically significant in control and AF samples. The heatmap of the DEGs is shown in Fig. 1, which indicated that the expression of identified DEGs could correctly distinguish the two types of samples.

### Differences in clinical characteristics between patients with and without ERAF

Overall, as is shown in Table 1, 65 patients who underwent RFCA were included, among whom, 45 without ERAF were defined as the control group, and 20 individuals with ERAF were defined as the study group who all occurred in the first 3 months. All RFCA patients had no complications such as pericardial tamponade and left atrial esophageal fistula. The patients comprised 44 males (67.7%) and 21 females (32.3%), with a mean age of 59.2 ± 11.2 years. The baseline characteristics, including body mass index (BMI), combined with basic diseases were not significantly different between the two groups. TRPV2 expression in PBMC samples analyzed by real-time PCR revealed an obvious downregulation in patients without ERAF compared to the ERAF group (0.5 [0.3, 0.7] vs. 0.9 [0.6, 1.1]). However, TRPV2 expression of the LRAF group was not significantly different compared to that of the without LRAF group (*p* > 0.05). In terms of the indicators of cardiac color Doppler ultrasound, the LA area of the ERAF group was higher than without ERAF group, although not statistically significant. The mitral flow diagram consists of the peak E, representing early filling, and peak A, representing late filling. The indicator of E/A < 1 reflected left ventricular diastolic dysfunction and increased LA pressure. As expected, the number of E/A < 1 after RFCA in the no ERAF group was markedly lower compared to that of the ERAF group (9 [25%] vs. 13 [76.5%]; *p* = 0.001), indicating that RFCA could alleviate LA pressure after sinus rhythm conversion. Due to the small number of postoperative cardiac color Doppler ultrasounds, larger sample data are needed to support this result. PWD has been reported to be related to ERAF or LRAF. In line with this, we found that the PWD in the ERAF group was significantly higher than no ERAF group (33 [25.5, 43] vs. 21 [11, 31]; *p* = 0.01).

**Table 1:** Baseline characteristics of patients with and without ERAF

	No ERAF	ERAF	Total	χ2/t/z	*p* value
	N = 45	N = 20	N = 65		
Male, n (%)	30 (66.7)	14 (70)	44 (67.7)	0.07	0.79
Age, years	58.3 ± 10.5	61.4 ± 12.1	59.2 ± 11. 2	1.2	0.29
BMI, kg/m^2^	24.2 ± 2.5	23.8 ± 3.8	24.1 ± 2.9	0.28	0.6
Systolic blood pressure(mmHg)	126.7 ± 22.8	131.1 ± 15.6	128.1 ± 20.7	0.61	0.42
Diastolic blood pressure(mmHg)	78.5 ± 11.7	80.6 ± 9.2	79.1 ± 10. 2	0.48	0.49
Heart rate(bpm)	81.5 (74,79)	75 (62,87.5)	80 (72.5,90)	− 1.05	0.29
Coronary artery disease, n (%)	9 (20)	4 (20)	13 (20)	< 0.01	1
Hypertension, n (%)	23 (51.1)	12 (60)	35 (53.8)	0.44	0.51
Diabetes mellitus, n (%)	7 (15.6)	6 (30)	13 (20)	1.02	0.31
Previous stroke or TIA, n(%)	15 (33.3)	9 (45.0)	24 (36.9)	0.81	0.37
History of thyroid disease, n (%)	7 (15.6)	2 (10)	9 (13.8)	0.04	0.83
*Type of AF*					
Paroxysmal, n (%)	19 (42.2)	9 (45.0)	28 (43.1)	0.04	0.84
Persistent, n (%)	26 (57.8)	11 (55.0)	37 (56.9)		
AF duration, months	1.56 (0.6,24.6)	3.8 (1.6,23.8)	1.8 (0.7,24.5)	− 0.99	0.32
RFCA time, mins	231 (215,280)	214 (181,270)	230 (197,280)	− 1.49	0.14
CHA2DS2-Vasc score	3.0 (2.0,4.0)	2.5 (1.0,4.0)	2.0 (1.0,3.0)	− 1.62	0.11
Smoker, n (%)	12 (26.7)	5 (25.0)	17 (26.2)	0.2	0.89
Drinking, n (%)	7 (15.6)	2 (10.0)	9 (13.8)	0.04	0.83
*Medications during blanking period*					
Amiodarone, n (%)	36 (80.0)	15 (75.0)	51 (78.5)	0.02	0.9
Beta blocker, n (%)	19 (42.2)	9 (45.0)	28 (43.0)	0.44	0.84
ACEi/ARB, n (%)	16 (35.6)	10 (50.0)	26 (40.0)	1.2	0.27
Warfarin/Rivaroxaban n (%)	45 (100)	20 (100)	65 (100)	–	–
Statin drugs, n(%)	29 (64.4)	16 (80)	45 (69.2)	1.57	0.21
*Echocardiographic parameters*					
Left atrial area,cm^2^	16.3 ± 3.9	18.0 ± 3.1	16.9 ± 3.7	3	0.09
Left ventricular end diastolic diameter,mm	47.8 ± 5.7	48.5 ± 4.5	48.0 ± 5.4	0.47	0.64
LVEF, %	65 (60,70)	68 (63.3,70.5)	65 (60.5,70)	− 1.21	0.23
E/A < 1 before RFCA,n(%)	22 (61.1)	15 (88.2)	37 (69.8)	2.85	0.09
E/A < 1 after RFCA,n(%)	9 (25)	13 (76.5)	22 (41.5)	10.57	0.001
*3D CT 0f heart and pulmonary vein*					
LAV(mL)	95.2 (62.7,119.2)	94.2 (69.9,121.7)	97.4 (66.7,122.7)	− 0.95	0.34
BSA(m2)	0.8 (0.8,0.9)	0.8 (0.7,0.9)	0.8 (0.8,1.0)	− 0.14	0.89
LAVI:LA volume/BSA (mL/m2)	107 (73.1,143.2)	99.8 (83.5,185.8)	102.1 (74.8,143.3)	− 0.95	0.515
Left superior PV diameter (mm)	10.2 (7.0,12.8)	10.4 (7.7,13.6)	10.4 (7.5,12.9)	− 0.24	0.82
Left inferior PV diameter (mm)	8.4 (6.6,11)	9.3 (7.6,11.4)	8.6 (7.2,11.3)	− 0.87	0.38
Right superior PV diameter (mm)	9.4 (7.0,12.3)	9.8 (8.4,11.9)	9.4 (7.5,12.2)	− 0.07	0.94
Right inferior PV diameter (mm)	9.8 (7.5,13)	9.9 (7.2,13.8)	9.5 (7.4,13.1)	− 0.03	0.98
*ECG after RFCA*					
PWD (ms)	21 (11,31)	33 (25.5,43)	25.5 (11.3,39.8)	− 2.54	0.01
mPWD(ms)	90 (81.5,97)	94 (78.3,96.3)	92.3 (77.9,100.3)	− 0.59	0.56
*Blood test index*					
WBC(× 10^9^/L)	6.6 (5.4,7.9)	6.0 (5.1,7.9)	6.3 (5.3,7.9)	− 0.85	0.4
NE(× 10 ^9^ /L)	3.9(3.0,5.2)	3.2 (2.4,5.0)	3.7 (2.8,5.2)	− 1.02	0.31
LYM(× 10 ^9^ /L)	2.0 (1.7,2.2)	1.8 (1.4,2.2)	1.9 (1.6,2.2)	− 0.8	0.43
HB(g/L)	142.4 ± 18.1	141.8 ± 14.4	142.2 ± 16.9	0.02	0.88
PLT(× 10 ^9^ /L)	229.9 ± 60.5	226.7 ± 61.9	228.9 ± 60.5	0.04	0.84
TSH(mIU/L)	1.4 (0.8,2.3)	1.8 (1.1,3.2)	1.7 (0.9,2.7)	− 1.68	0.09
FT3(pmol/L)	5.3 (4.7,5.6)	5.2 (4.4,5.5)	5.2 (4.7,5.6)	− 0.7	0.48
FT4(pmol/L)	16.9 ± 3.1	15.2 ± 3.1	16.4 ± 3.1	− 1.96	0.05
PT(s)	11.2 (10.6,11.9)	11.8 (11.1,13.6)	11.3 (10.8,12.9)	− 1.96	0.05
APTT(s)	27.4 (25.8,29.7)	29.3 (26.6,32)	28.1 (26,30.9)	− 1.32	0.19
D-Dimer(FEU)	0.3 (0.2,0.5)	0.3 (0.2,0.5)	0.3 (0.2,0.5)	− 0.79	0.43
CREA(umol/L)	73.3 (62,84.6)	73.7 (60.8,85.1)	73.3 (62,86.3)	0.001	0.98
UA(umol/L)	371(308,436.5)	372.5(281,412)	371(304,427)	0.59	0.45
TRPV2 mRNA expression before RFCA	1.0 (1.0,1.0)	1.0 (1.0,1.0)	1.0 (1.0,1.0)	− 0.02	0.98
TRPV2 mRNA expression after RFCA	0.5 (0.3,0.7)	0.9 (0.6,1.1)	0.6 (0.3,0.9)	− 3.58	< 0.001

ACEi angiotensin-converting-enzyme inhibitors; ARB angiotensin II receptor type 1 blockers; BMI body mass index; LAV left atrial volume; BSA body surface area; mPWD mean P wave dispersion;UA Uric acid; CREA creatinine; ERAF early recurrence of atrial fibrillation; LVEF left-ventricular ejection fraction; LAVI: LAV/BSA. *p* values in italics are statistically significant at a level of *p* < 0.05

### Correlation analysis

**Fig. 2:**
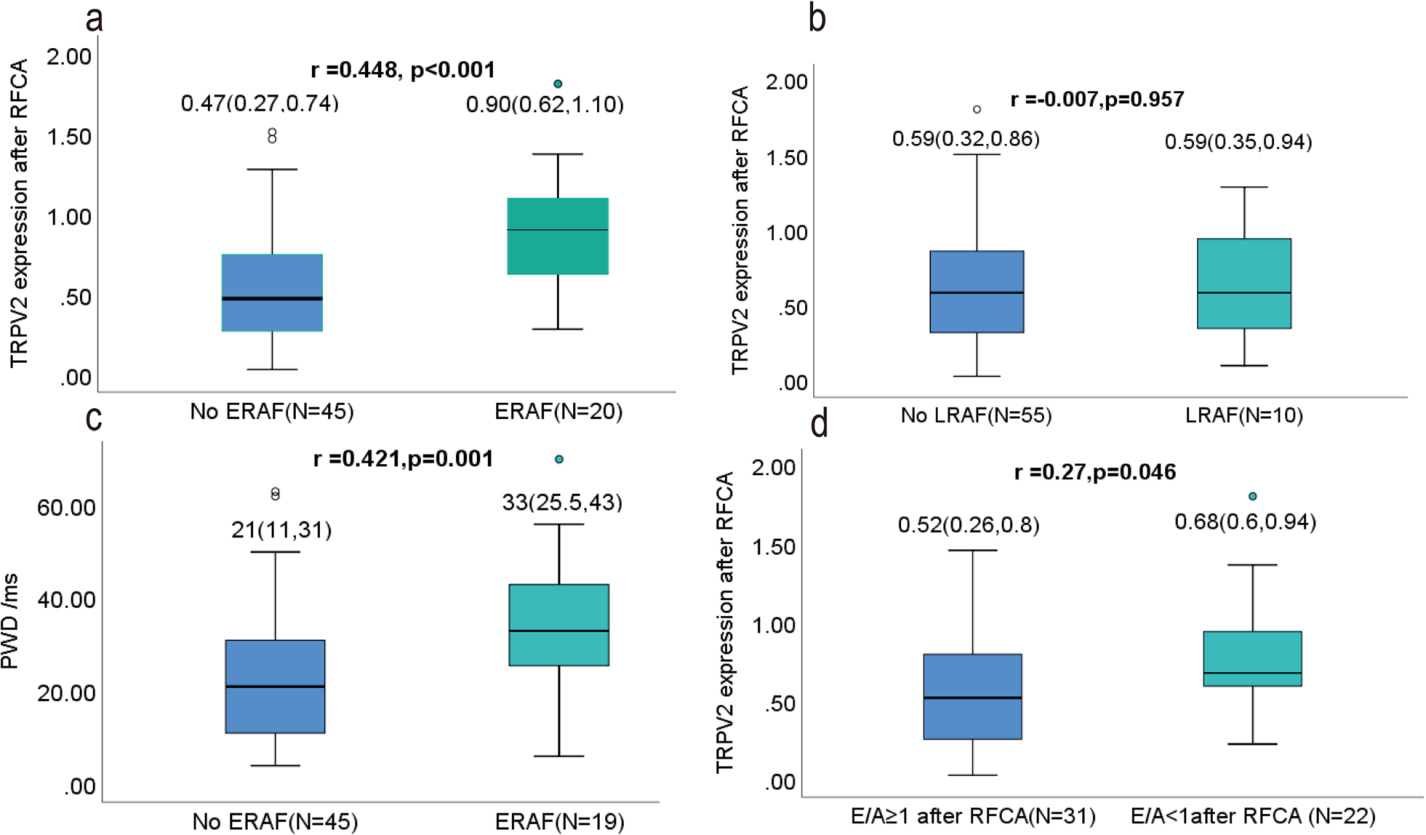
Study on the Spearman correlation between factors and ERAF. **a** the level of TRPV2 mRNA after RFCA versus ERAF (r = 0.45, *p* < 0.001), **b** LRAF versus ERAF(r = − 0.007, *p* = 0.96), **c** PWD from ECG after RFCA versus ERAF (r = 0.42, *p* = 0.001), **d** E/A after RFCA versus TRPV2 mRNA expression after RFCA (r = 0.27, *p* = 0.046)

Spearman’s Rho test was used to analyze the correlations between ERAF and related variables (Fig. 2). There were significant Spearman correlations between ERAF and TRPV2 mRNA expression after RFCA (r = 0.45, *p* < 0.001), PWD (r = 0.42, *p* = 0.001). There were moderately strong Spearman correlations between TRPV2 mRNA expression after RFCA and E/A after RFCA (r = 0.27, *p* = 0.046). Patients with AF after RFCA could improve LV diastolic dysfunction and LA pressure, which may contribute to suppress the expression of TRPV2 mRNA. However, the Spearman correlation between TRPV2 mRNA expression after RFCA and LRAF was not significant (*p* = 0.96).

### Predictors of ERAF after RFCA

**Fig. 3:**
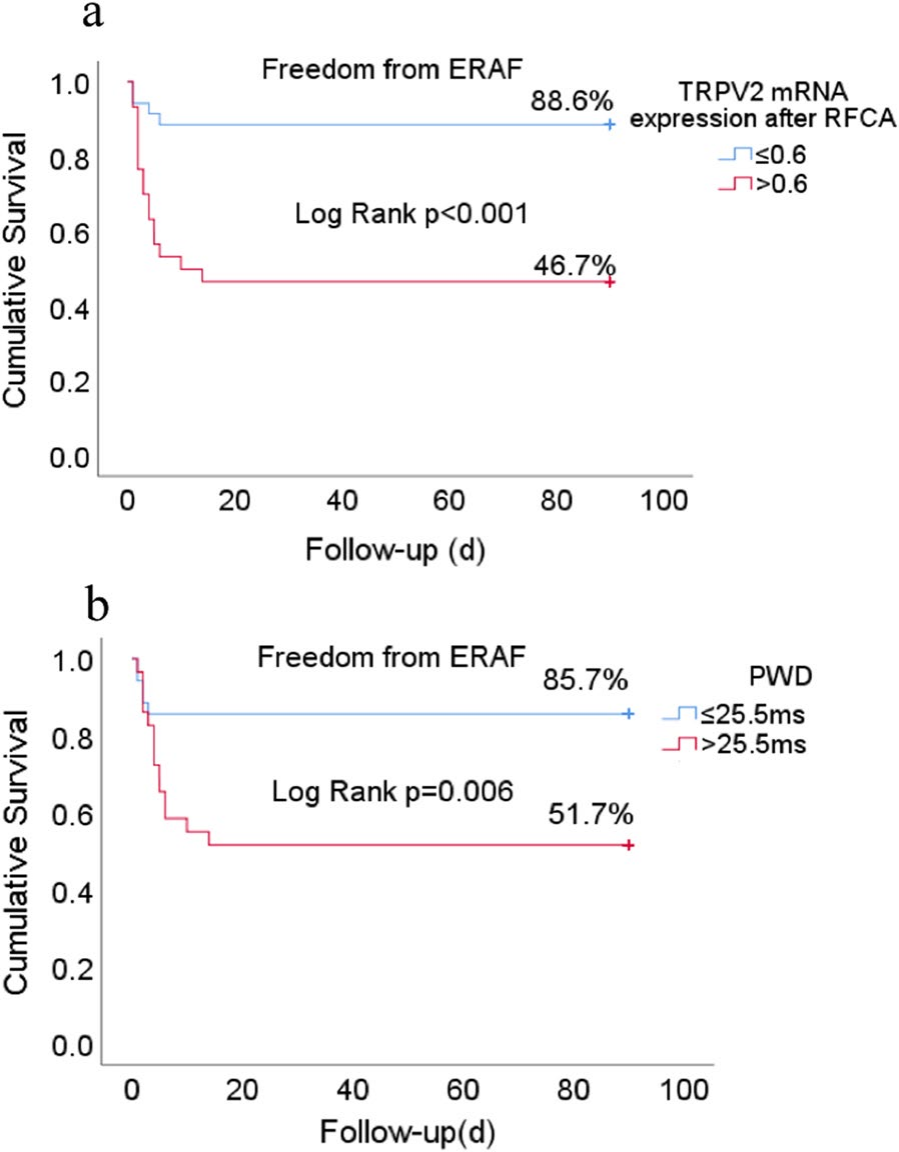
Survival analysis using the Kaplan–Meier method and log-rank test to compare clinical outcomes. **a** Freedom from ERAF by TRPV2 mRNA expression after RFCA with/without > 0.6; **b** Freedom from ERAF by PWD after RFCA with/without > 25.5 ms. Caption: Time zero indicates day of procedure

The results of univariate and multivariable binary logistic regression model analyses are summarized in Table 2. Although the factor of E/A < 1 after RFCA had a statistically significant association with ERAF on univariate analysis, it was not selected for multivariable analysis because of the number of missing values. On multivariate analysis, both TRPV2 mRNA expression after RFCA (odds ratio [OR], 3.83; 95% confidence interval [CI], 1.64–8.93; *p* = 0.002) and PWD (OR, 1.09; 95%CI, 1.02–1.16; *p* = 0.01) were independent positive predictors of ERAF. Taking the median TRPV2 mRNA expression after RFCA (0.6) and PWD (25.5 ms), survival analysis using the Kaplan–Meier method and log-rank test showed significant differences in ERAF after RFCA in both. As shown in Fig. 3a, Kaplan–Meier analysis showed a lower risk of ERAF in 4 of 35 patients (11.4%) with TRPV2 mRNA expression after RFCA ≤ 0.6 and 16 of 30 patients (54.3%) with TRPV2 mRNA expression after RFCA > 0.6 (log-rank *p* < 0.001). As shown in Fig. 3b, Kaplan–Meier analysis showed a higher risk of ERAF in 14 of 29 patients (48.3%), with an ECG of PWD > 22.5 ms, and 5 of 35 (14.3%) with a PWD ≤ 22.5 ms (log-rank *p* = 0.006).

**Table 2:** Binary logistic regression model analysis for predictor s of ERAF

	Univariate regression analysis			Multivariable regression analysis		
	Odds ratio	95% confidence interval	*p* value	Odds ratio	95% confidence interval	*p* value
Paroxysmal AF	1.12	(0.39,3.23)	0.84			
Left atrial area, cm^2^	1.14	(0.09,1.14)	0.09	1.23	(0.99,1.53)	0.06
PWD (ms)	0.96	(0.92,0.99)	0.023	1.09	(1.02,1.16)	0.01
TRPV2 mRNA expression after RFCA	2.76	(1.44,5.27)	0.002	3.83	(1.64,8.93)	0.002
TSH(mIU/L)	1.19	(0.27,1.63)	0.27			
FT4(pmol/L)	0.89	(0.75,1.06)	0.21			
PT(s)	1.05	(0.89,1.24)	0.54			

PWD: P wave dispersion; ERAF: early recurrence of atrial fibrillation; *p* values in italics were statistically significant at a level of *p* < 0.05

### Defining cut-off values

**Fig. 4:**
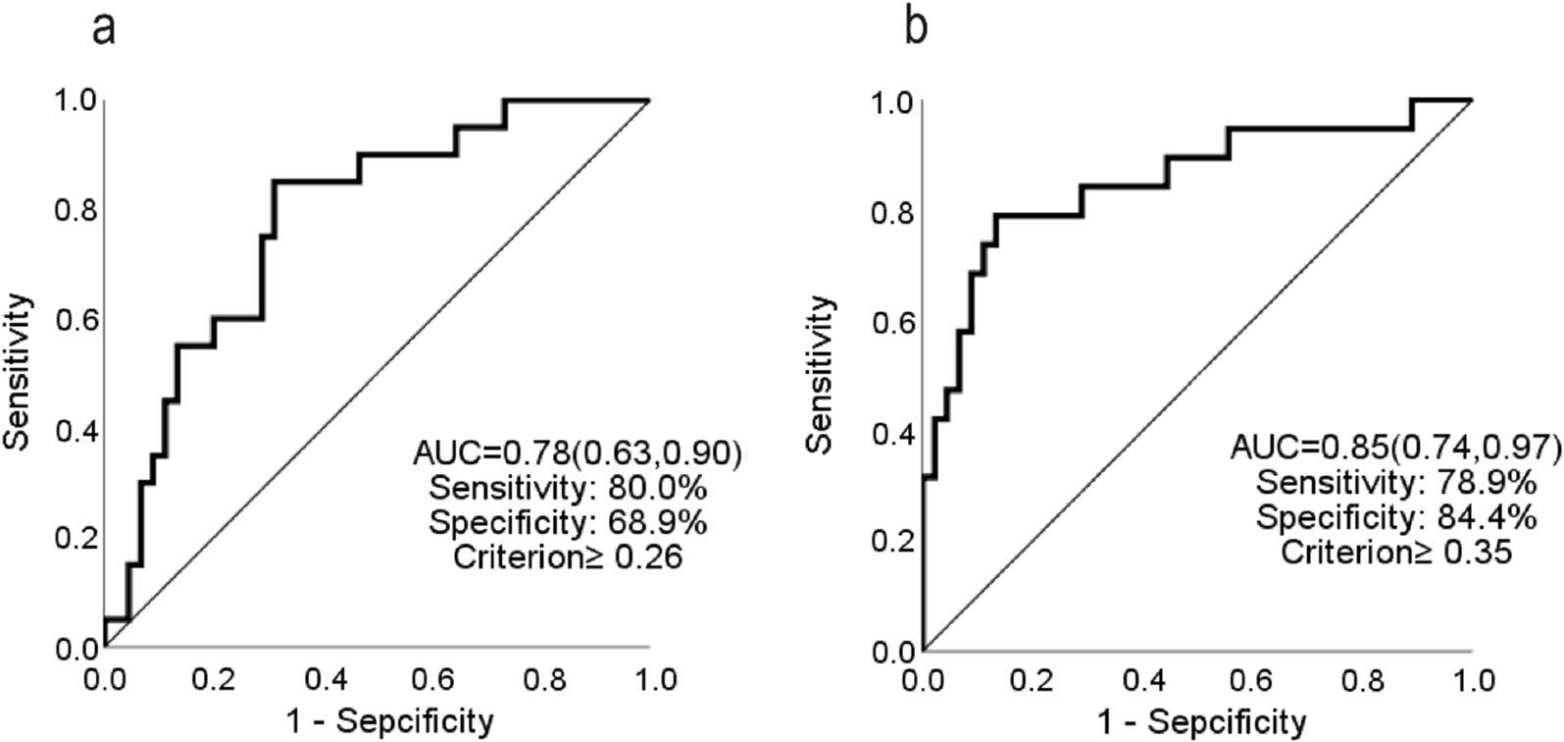
Receiver operating characteristic (ROC) curve for the prediction of ERAF. Model 1 **a** used single factor of TRPV2 mRNA expression after RFCA, indicting the ROC curve is 0.78 (*p* < 0.001). Model 2 **b** used two factors of TRPV2 mRNA expression and PWD after RFCA, indicting the ROC curve is 0.85 (*p* < 0.001)

ROC curves were used to obtain the optimal cut-off values through predictors of TRPV2 mRNA expression after RFCA (model 1) and combined TRPV2 mRNA expression after RFCA and PWD (model 2). The area under the ROC curve of the model 1 curve was 0.78 (95% CI: 0.633–0.897, *p* < 0.001). We chose the optimal cut-off value of ≥ 0.26 according to Youden’s index; the sensitivity and specificity of the optimal cut-off value were 80% and 68.9%, respectively (Fig. 4a), showing that the level of TRPV2 mRNA expression after RFCA ≥ 0.26 was the strongest predictive value for ERAF. The area under the ROC of the model 2 curve was 0.85 (95% CI: 0.737–0.965, *p* < 0.001). We chose the optimal cut-off value ≥ 0.35 according to Youden’s index; the sensitivity and specificity of optimal cut-off value were 78.9% and 84.4%, respectively (Fig. 4b).

### Pathway enrichment analysis

**Fig. 5:**
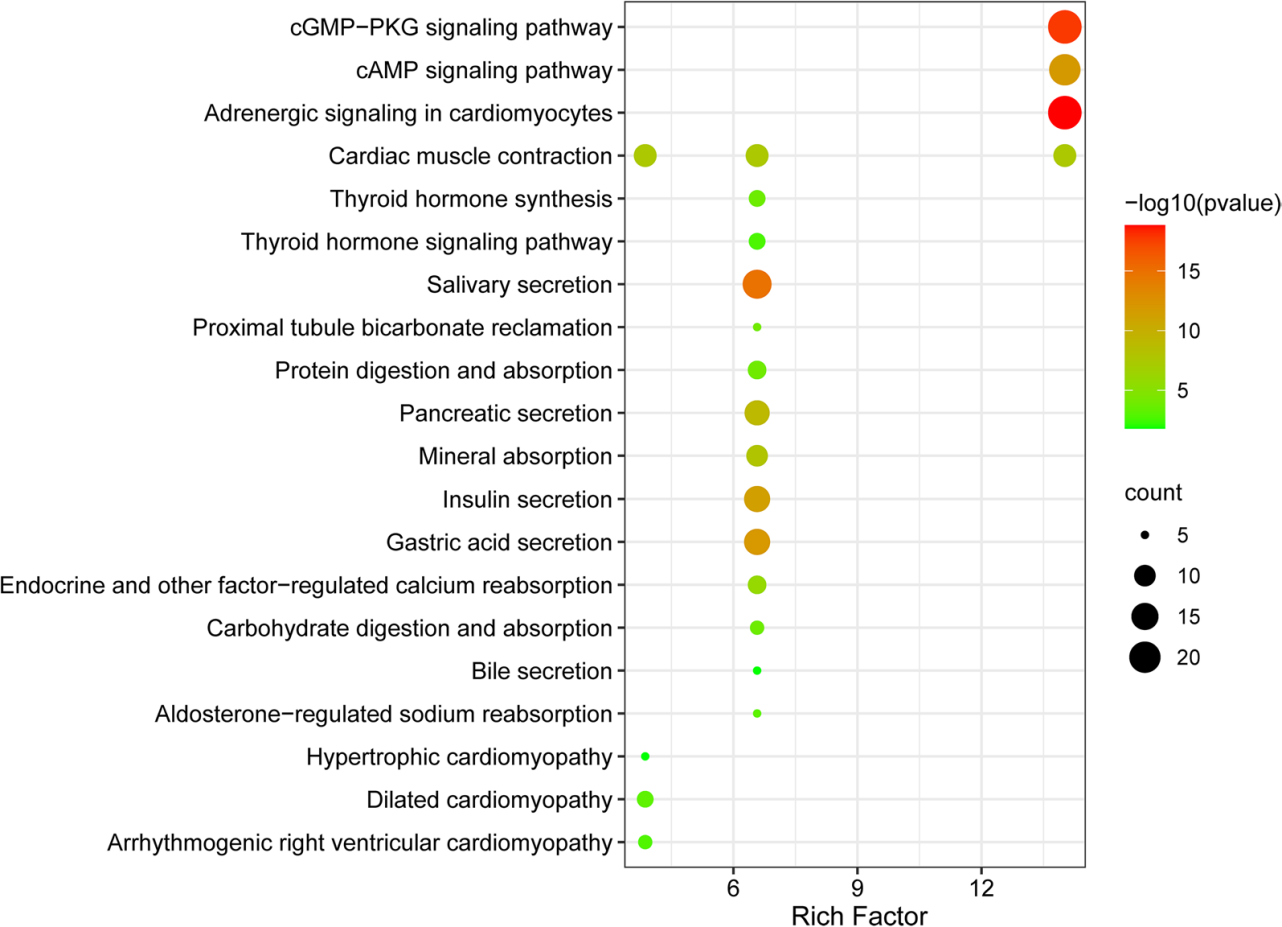
Scatter plot of enriched KEGG pathways statistics. Rich factor is the ratio of the differentially expressed gene number to the total gene number in a certain pathway. Q-value is corrected P-value ranging from 0 to 1. The color and size of the dots represent the range of the Q-value and the number of DEGs mapped to the indicated pathways, respectively. Top 22 enriched pathways are shown in the figure

The pathogenesis of the AF-mediated TRPV2 signaling pathway has not yet been established; thus, to investigate possible signaling pathways, we mapped the KEGG database through 124 genes, namely 96 cardiac ion channels and accessory proteins [18], and 28 human TRP channels [19]. As a result (Fig. 5), we found that the identified DEGs were significantly enriched in 41 KEGG pathways (Q-value < 0.01). DEGs were highly clustered in several signaling pathways, including the “cGMP-PKG signaling pathway,” “cyclic adenosine monophosphate (cAMP) signaling pathway,” and “adrenergic signaling in cardiomyocytes,” indicating that TRPV2 may perform its function through these pathways.

## Discussion

### Main findings

To the best of our knowledge, this is the first study to analyze ERAF after RFCA and its association with the expression profile of TRPV2. According to our results, a significant downregulation in the expression of peripheral TRPV2 was associated with a lower risk of ER, indicating that TRPV2 may represent a novel predictor of the early phase after successful radiofrequency ablation.

### Ca^2+^-handling dysregulation and pathophysiological mechanism of ERAF

It was found that TRPV2 mRNA was significantly upregulated in LA tissue [17] from patients with AF through DEG analysis, indicating that TRPV2 involved in pathophysiological mechanism of AF. TRPV2 plays an important role in calcium homeostasis [20]. Ca^2+^-handling abnormalities mediated by electrical remodeling are common in atrial cardiomyocytes of patients with AF, as well as in patients with risk factors and comorbidities promoting the occurrence and maintenance of AF [21]. Greiser et al. reported that altered Ca^2+^ signaling during AF progression consists of three phases: Ca^2+^ overload, remodeling, and a steady state [22]. Ca^2+^-handling dysregulation can also promote beat-to-beat alternation in action potential duration (APD), which is produced most readily in patients with PerAF, less readily in ParAF, and even less in sinus-rhythm controls [23]. In dogs, AF-induced remodeling delays the recovery of transient cellular Ca^2+^, which causes increased magnitude and spatial dispersion of susceptibility to Ca^2+^—and repolarization-alternans, and finally leads to reentrant rotor formation and enhanced vulnerability to the initiation and maintenance of AF [24].

Moreover, Ca^2+^ waves triggered by L-type Ca^2+^ channels at the cell boundary might activate Ca^2+^-overloaded sarcoplasmic reticulum (SR) sites at the cardiomyocyte center, leading to large triggered Ca^2+^ waves (TCWs) during the AP. This causes delayed afterdepolarizations (DADs) to reach the threshold of ectopic lesion discharge, and sustained delayed depolarization potential can also cause AF [25]. In addition, spontaneous Ca^2+^ release-promoting triggered activity is considered an important mechanism of AF initiation [26]. A trial in isolated rabbit atrial myocytes suggested that silencing Ca^2+^ signaling through failure of subcellular-propagated Ca^2+^ release is a protective mechanism against the massive Ca^2+^ overload that occurs during chronic AF [22]. The recovery of sinus rhythm in patients with AF after RFCA is closely related to the change in Ca^2+^ flow in cardiomyocytes. Catheter ablation offers a greater chance of achieving and maintaining SR; however, it is unclear how long the benefits are sustained, and whether early restoration of SR will result in better long-term outcomes [27]. Abnormal Ca^2+^ evokes PV triggers after RFCA, and the regulatory proteins of Ca^2+^ handling are important contributors to an SR Ca^2+^ overload, diastolic membrane instability, and AF occurrence [7]. Yue et al. [28] examined the changes in the miRNA of 90 patients with AF before and 3 months after RFA and found that the expression of miR-377-5p increased 4.27-fold before RFA and decreased 102.89-fold after RFA in patients with AF. This miRNA plays a key role in the regulation of CACNA1C expression, reduction in the L-type Ca^2+^ current (ICaL), and shortening of APD and effective refractory period (ERP). The Ca^2+^ loading leads to ICaL (Ca^2+^) channel inactivation, reduces the APD, and causes atrial structural and electrical remodeling, which may set off an AF trigger in both the PV and non-PV areas, initiating a vicious cycle. Therefore, the key to reduce the recurrence of atrial fibrillation caused by calcium overload is successful PVI. It was demonstrated that AI-guided catheter ablation is associated with high effectiveness and good safety of RFCA [29]. In this study, all patients underwent ablation under the guidance of AI, and the recurrence rate of ERAF was only 38.5%, significantly lower than that reported in other studies [2, 3]. Actually, the great majority of the recurrent cases are from PerAF. Thus, the ablation strategy for PerAF should be further explored. Yao et al. [30] found that the ablation procedure in Group I (PVI + LA roof line + LA anterior wall line) had higher success rate than Group II (PVI + LA roof line). Moreover, PVI combined roof and mitral isthmus line for PerAF ablation, can contribute to alteration of AF wavelet propagation, elimination of spectral components, reduction of excitable LA myocardial mass and attenuation of vagal innervation [31, 32]. Therefore, PVI combined LA roof and mitral isthmus line ablation are routinely selected for patients with PerAF in our AF center.

### Predictive value of TRPV2

Recently, it was revealed that LA pressure was increased during the onset of AF, which was linked to TRPV2 expression [33]. Animal studies have further confirmed that the LA pressure is increased, and the induction rate of AF is also increased and easy to maintain [34] and can also increase ectopic activation of pulmonary vein [35], suggesting that the increase in LA pressure plays an important role in the recurrence of AF. The level of TRPV2 expression in cardiomyocytes was 10 times higher than that in other cells, and the activation of TRPV2 channels could cause an increase in intracellular Ca^2+^ and lead to dilated cardiomyopathy [36]. Elevated LA pressure was closely associated with electroanatomic remodeling of the LA and was an independent predictor of recurrence after AF ablation [37]. The restoration of sinus rhythm after RFCA of AF can reduce the LA pressure, which may explain the decrease in TRPV2 expression after RFCA. Our study showed for the first time that TRPV2 from PBMCs could predict the ER of patients with AF after RFCA. Previous studies have confirmed that TRPV2 expression in human PBMCs is highly consistent with that of cardiomyocytes, which may represent the changes in the gene regulation of the myocardium. The data obtained may demonstrate whether the elevated numbers of TRPV2-expressing monocytes/macrophages in the periinfarct zone are associated with modified numbers of TRPV2-expressing PBMCs [20, 38], and thus enable better characterization of TRPV2 as a novel marker of early phase after RFCA.

According to our study, it was found that there was no difference in the expression level of TRPV2 between ParAF and PerAF. In addition, the level of TRPV2 measured in 29 healthy people was almost equal to that of the above patients (Additional files 1, 2). Thus, we could infer that sinus rhythm before RFCA had no significant effect on TRPV2 expression, while the expression level would have decreased significantly in the without ER group after achieving sinus rhythm. As TRPV2 is a transient protein, it could sensitively reflect the change process from AF to sinus rhythm by RFCA but was unable to determine whether AF was present before RFCA and whether it developed LRAF after RFCA. Furthermore, the average recurrence time of these 25 patients with ERAF was 3.5 days according to our study. It was unexpected that there was already a difference in the expression level of TRPV2 when all patients had restored sinus rhythm by using bedside surface ECG. Indeed, the success of the procedure could not be determined simply based on the postprocedural bedside ECG. According to the 2020 ESC Guidelines for the diagnosis and management of atrial fibrillation [1], AF is divided into clinical AF, atrial high-rate episode (AHRE), and subclinical AF (SAF). SAF is also called asymptomatic AF, and the prevalence of SCAF may be seriously underestimated, which is associated with increased cardiovascular and all-cause mortality and significant stroke risk SAF [39]. Asymptomatic AF is detected by implantable and wearable cardiac devices which is too expensive and inconvenient to widely be used in clinical. Hindricks et al*.* reported that more than 50% of patients had symptomatic and asymptomatic AF before RFCA, while only 38% of patients with AF were accurately identified; within the first 3 months after RFCA, asymptomatic arrhythmia recurrence (mainly ERAF) reached to 38% [40]. Therefore, how to identify the asymptomatic ERAF is an urgent clinical problem. In this case, on the one hand, we reduce the recurrence rate by improving the technical level of RFCA; on the other hand, we identify postoperative inpatients with asymptomatic ERAF through short-term ECG monitoring, pulse measurement, and auscultation. For discharged patients, we usually advise patients to palpate their pulse rhythm every day, and these patients will return to the clinic for 1–2 times every month to further identify whether there is asymptomatic ERAF. Our current study is also designed based on the background of the difficulties encountered in the diagnosis of ERAF in clinic and actively exploring predictive value of TRPV2. Even if AF is not captured on the body surface ECG, it is likely that patients with ERAF had AHRE and SAF, causing upregulation of TRPV2. This hypothesis could also explain why there was no significant difference in TRPV2 expression between patients with PerAF and ParAF before RFCA. Nevertheless, it is still necessary to diagnose AHRE and SAF by cardiovascular implantable electronic device (CIED) or wearable ECG detector to obtain direct evidence of an association with high expression of TRPV2. In addition, postoperative PWD for predicting the recurrence of AF has attracted increasing attention [41, 42]. Previous studies showed a prolongation of the atrial conduction time as a result of atrial electrical remodeling, and a prolonged P wave duration was a momentous risk of the development of AF [43, 44]. Li et al. [45] demonstrated that PWD, maximum P-wave duration, P-wave terminal force in lead V1, PR interval prolongation can effectively predict AF recurrence after RFCA. This study also confirmed that TRPV2 mRNA expression combined with PWD after RFCA enhanced the predictive value from 0.78 to 0.85 according to the ROC curve.

DEG analysis suggested that TRPV1 and TRPV4 mRNA expression was consistent with TRPV2 in LA tissue. Therefore, we will explore the detection of TRPV1 and TRPV4 mRNA expression levels from PBMCs to identify whether they also have value in predicting ERAF after RFCA. Nevertheless, the role of TRPV2 channels in the pathogenesis of ERAF after RFCA remains unknown. KEGG mapping implies that several signaling pathways are highly enriched, which provides a possible research direction for the study of the mechanism of ERAF. Finally, TRPV2 signal regulation would provide a new idea for developing potential novel therapeutic targets for ERAF after RFCA.

## Limitations

First, it is undeniable that the sample size of the ER group was relatively small and unequal to that of the without ER group, which may lead to bias in statistical analysis. For discharged patients, although we can detect asymptomatic ERAF as much as possible through the patient's regular follow-up ECG examination and their own evaluation of pulse rhythm, there may still be a possibility of missing diagnosis of asymptomatic AF. Second, we found no association between left atrial size, AF duration, or type of AF and ER occurrence, while the correlation of both had been confirmed in previous studies [3]. In addition to the statistical bias caused by small sample size, in clinical work, the operators give priority to patients with smaller left atria, shorter duration of PerAF, and other factors, which will also reduce the differences between groups to a certain extent. Third, this study was a single center study, which limited the generalizability of our results. Larger samples with more reliable follow-up methods are needed to clarify the relevance and optimal management of ER and LR.

## Conclusions

We assume that downregulated expression of peripheral TRPV2 appears in patients without early recurrence of AF after radiofrequency ablation. Therefore, TRPV2 may represent a novel predictor of the early phase after successful radiofrequency ablation.

## Supplementary Information

**Additional file 1.** Maps of RFCA.

**Additional file 2.** TRPV2 mRNA expression before RFCA of AF patients and health individuals.

## Data Availability

The datasets used and/or analysed during the current study are available from the corresponding author on reasonable request.

## Abbreviations

TRPV2: Transient receptor potential vanilloid 2
PerAF: Persistent atrial fibrillation
ParAF: Paroxysmal atrial fibrillation
PBMCs: Peripheral blood mononuclear cells
ERAF: Early recurrence of atrial fibrillation
GSEA: Gene set enrichment analysis
ROC: Receiver operating characteristic
PWD: P wave dispersion
PVI: Pulmonary vein isolation
LR: Late recurrence
DEGs: Differentially expressed genes
KEGG: Kyoto Encyclopedia of Genes and Genomes
DAVID: Database for annotation, visualization and integrated discovery
AHRE: Atrial high-rate episode
SAF: Subclinical AF
AI: Ablation index
