# Supplementary material for: Predictive value of TRPV2 expression from peripheral blood mononuclear cells on the early recurrence of atrial fibrillation after radiofrequency catheter ablation
Source: BMC Cardiovasc Disord. 2022 Dec 13;22:546. doi: 10.1186/s12872-022-02992-0 (PMC9746099; doi:10.1186/s12872-022-02992-0)
Supplement: Supplementary file 1 — Additional file 1. Maps of RFCA. [file 12872_2022_2992_MOESM1_ESM.pdf]

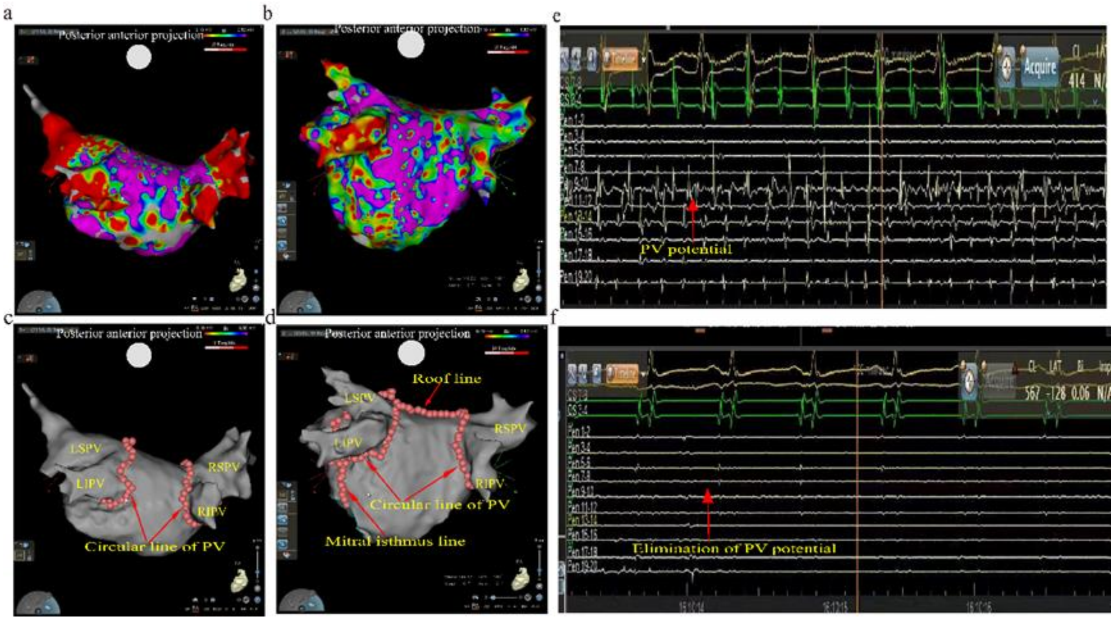

Fig.S1 Maps of RFCA. **a** Matrix mapping before ablation of ParAF; **b** Matrix mapping before ablation of PerAF; Matrix mapping is to better reflect the voltage distribution of LA and PV, and the color from red to purple represents the voltage from low to high. **c** Ablation target map of ParAF. A circular linear ablation path was performed at the antrum of the left and right PV. **d** Ablation target map of PerAF. The roof and mitral isthmus line was also performed besides circular linear ablation. **e** The PV potential is extremely obvious before RFCA. **f** The elimination of PV potential is due to PVI successfully. LSPV: Left superior pulmonary vein; LIPV: Left inferior pulmonary vein; RSPV: Right superior pulmonary vein; RIPV: Right inferior pulmonary vein; PerAF: Persistent atrial fibrillation; ParAF: Paroxysmal atrial fibrillation; PVI: Pulmonary vein isolation.
